# Enhancing Communication and Patient Care: The Reverse Stethoscope Technique

**DOI:** 10.7759/cureus.22069

**Published:** 2022-02-09

**Authors:** Radhika Sharma, Swetha R Nuthulaganti, Ravindra P Maharaj

**Affiliations:** 1 Internal Medicine, University of Florida College of Medicine – Jacksonville, Jacksonville, USA; 2 Internal Medicine: Geriatrics and Palliative Care, University of Florida College of Medicine – Jacksonville, Jacksonville, USA

**Keywords:** hearing-aid, health disparity, effective communication, deafness, reverse stethoscope technique, communication

## Abstract

Patients with hearing loss have increased difficulties while navigating communication, especially complex conversations that take place in healthcare settings. Patients with hearing disabilities may often find themselves in noisy reception areas or busy emergency departments where their confidentiality is compromised for the sake of communication, resulting in suboptimal care. The reverse stethoscope technique is a tool that healthcare professionals can use to enhance communications to optimize patient care and maintain confidentiality. We describe a case of an 83-year-old male with bilateral hearing loss whose hearing disability became a major obstacle in his access to healthcare in the inpatient setting. With the help of the reverse stethoscope technique, the patient was able to understand his hospital course, diagnosis, and treatment options and was able to actively participate in making an informed decision.

## Introduction

Communication is a foundational aspect of patient-centered care. Effective patient-provider communication allows for the transfer of information regarding medical symptoms, diagnosis, and treatment options and facilitates shared decision-making guided by patients’ values [1]. With strong communication skills, providers can help patients navigate the complexities of their medical illness and ultimately improve quality and cost of care and patient satisfaction [1, 2].

Patients with hearing disabilities are unable to navigate the healthcare system as easily as their hearing-able counterparts. The 2019 Global Burden of Disease Study found that hearing loss is the third leading cause of disability globally; about 1.57 billion people are estimated to suffer from it. In the United States, it is estimated that about one-third of adults from ages 61 to 70 and more than 80 percent of those older than 85 live with some form of hearing loss [2-4]. The association between hearing disability and age is evident - the prevalence of hearing loss is predicted to double with every 10-year increase in age, and it is expected that if more of the population continues to age, nearly everyone will suffer from some degree of hearing disability [3].

Hearing loss directly impacts patients’ abilities to engage and maintain oral communication and is associated with overall negative health outcomes, increased risk of hospitalization, and mortality [1]. Communicating in healthcare settings is difficult for patients with hearing loss as both inpatient and outpatient healthcare settings can have high ambient noise which can greatly hinder patients’ hearing capabilities [1, 5]. Additionally, medical communication is often convoluted with foreign terms, and difficulty navigating these conversations leads to increased patient embarrassment and anxiety in settings that may already have high levels of environmental and personal stressors. Hearing loss leads to isolation: patients may not be able to converse on the telephone or through telemedicine, limiting their ability to book appointments or to receive care virtually [5].

Despite data on the increasing prevalence of hearing loss amongst an aging population, literature that addresses the serious implications of hearing disabilities on the quality of healthcare is scarce [1, 2, 4].

## Case presentation

An 83-year-old male with medical history pertinent for bilateral hearing loss presented to the urgent care clinic for a post-discharge follow-up visit. The patient was recently admitted for post obstructive pneumonia with image findings concerning for malignancy. During the admission, computed tomography angiography of the chest showed obstruction of the left lower segmental bronchus with associated atelectasis - secondary to either mucous plugging vs. underlying malignancy. Discharge documentation indicated that during a conversation to establish goals of care, the patient had indicated that he was not interested in any further workup for malignancy.

During the follow-up visit, the recent hospital admission and diagnoses were reviewed with the patient. Although the patient’s hearing aids were intact, it became clear that he was having difficulty with the conversation. The patient revealed that the batteries to his hearing aids had not been working for some time. Communication enhancement techniques such as effective positioning, clear and slow pronunciation, and body language were used to improve the conversation. Even with the use of enhancement techniques, the patient was unable to understand what was being communicated. Finally, the reverse stethoscope technique was employed: after patient consent, the earpieces of the stethoscope were cleaned and placed into his ears. Comfortable and effective placement was ensured. The provider then spoke quietly into the diaphragm of the stethoscope with slow and clear speech, using the tool as a microphone.

The reverse stethoscope technique aided with effective communication and ensured confidentiality. After reviewing his recent hospital admission and imaging results, the patient decided to pursue further workup for malignancy and stated that he would be willing to discuss the risk and benefits of associated therapy options.

## Discussion

Hearing loss is a medical disability and health disparity: individuals with a sensory deficit will systematically experience greater obstacles to healthcare and are therefore in a position where they may inevitably receive suboptimal care [1, 5]. Prior studies have shown that patients with hearing loss may experience more difficulties with comprehension and engagement in discussions with their providers leading to negative psychosocial, physical, and cognitive outcomes [1, 2, 4].

Healthcare professionals can help to further health equity or to help attain a higher level of health for those with hearing loss by being observant of their patients’ needs and adapting their communication skills to those of their patients [2, 5]. Providers should be diligent in identifying patients with hearing loss and once identified, providers can use a variety of strategies to improve communication. These strategies include proper positioning by facing the patient while speaking, using clear and slow pronunciation, using body language, and using written supplemental aids [2, 5]. Patients with hearing impairment often also use lipreading as a tool to navigate conversations [5-7].

The COVID-19 pandemic and widespread use of masks have made it increasingly difficult for patients with a hearing disability to communicate as they can no longer rely on the tool of lip-reading and facial expression [6, 7]. In such instances, the reverse stethoscope technique can be one cheap and easy method to improve the quality of care for some of our hearing-impaired patients while also maintaining confidentiality [8]. This aid was specifically helpful to our patient as he was unable to watch our mouth movements and use lip-reading cues as he usually did due to the use of masks as part of COVID-19 precautions.

Hearing loss severely limits the ways in which a patient can access healthcare and affects the quality of healthcare received. The purpose of this case report was to highlight one such case: because of his hearing disability, our patient was initially unable to engage in effective communication with his providers which led to a failed understanding of his medical diagnosis and rejection of a therapeutic option. With the help of the reverse stethoscope technique, the patient was able to comprehend his diagnosis and treatment options and was able to actively participate in making an informed decision regarding his care.

## Conclusions

Hearing loss is a health disparity as patients with hearing disabilities experience increased obstacles to access healthcare and are at an increased risk of receiving suboptimal care. Healthcare professionals can help to attain a higher level of care for those with hearing disabilities by being observant of their patients’ needs. The reverse stethoscope technique is an efficient and effective method to improve the quality of care for hearing-impaired patients while also maintaining patient-physician confidentiality in a busy healthcare setting.
